# Diminished humoral responses against and reduced gene expression levels of human endogenous retrovirus-K (HERV-K) in psoriasis

**DOI:** 10.1186/s12967-014-0256-4

**Published:** 2014-09-16

**Authors:** Rashmi Gupta, Henri-Alexandre Michaud, Xue Zeng, Maya Debbaneh, Sarah T Arron, R Brad Jones, Christopher E Ormsby, Douglas F Nixon, Wilson Liao

**Affiliations:** Department of Dermatology, University of California, 2340 Sutter Street, P.O. Box 0808, , 94143-0808 San Francisco, CA USA; Division of Experimental Medicine, University of California, San Francisco, USA; Equipe Immunité et Cancer, Institut de Recherche en cancérologie de Montpellier, 208 rue de Apothicaires, 34298 Montpellier cedex 5, France; Department of Dermatology, Guang’anmen Hospital, China Academy of Chinese Medical Sciences, Beijing, China; University of California, Irvine School of Medicine, 515 Spruce Street, San Francisco, CA USA; Department of Immunology, University of Toronto, Toronto, ON Canada; Centro de Investigaciones en Enfermedeades Infecciosas, Instituto Nacional de Enfermedades Respiratorias, Calz. de Tlalpan #4502, Colonia Sección XVI, Código postal 14080 México, DF MEXICO; Department of Microbiology, Immunology and Tropical Medicine, School of Medicine and Health Sciences, The George Washington University, Washington, DC USA

**Keywords:** Psoriasis, HERV-K, Immune response, Expression

## Abstract

**Background:**

Psoriasis is a multifactorial, chronic disease of skin affecting 2-3% of the world’s population. Genetic studies of psoriasis have identified a number of susceptibility genes that are involved in anti-viral immunity. Furthermore, physiological studies have also found an increase in anti-viral proteins in psoriatic skin. These findings suggest the presence of an anti-viral state in psoriatic skin. However, the triggers for this anti-viral cascade and its consequences for host immunity are not known. Endogenous retroviruses have previously been described in many autoimmune diseases including psoriasis.

**Methods:**

In the present study we examined the humoral immune response against human endogenous retrovirus-K (HERV-K) proteins and the cutaneous expression levels of multiple HERV-K genes in psoriasis patients and healthy controls.

**Results:**

In psoriatic sera we observed a significant decrease in IgM response against three HERV-K proteins: Env surface unit (SU), Env transmembrane protein (TM), and Gag capsid (CA) in comparison to sera obtained from blood bank healthy controls. A decrease in IgG response was also observed against CA. Furthermore, using quantitative RT-PCR we observed a decrease in the expression of HERV-K Env, Gag, Pol and Rec as well as ERV-9 genes in lesional psoriatic skin as compared to healthy skin.

**Conclusions:**

Together, our results suggest that the pro-inflammatory, anti-viral state in psoriasis is associated with diminished expression of HERV-K gene transcripts and a concomitant decrease in humoral responses to HERV-K. Our results indicate that a simple model where continuous, minimally changing HERV-K expression serves as an antigenic trigger in psoriasis might not be correct and further studies are needed to decipher the possible relationship between psoriasis and HERVs.

**Electronic supplementary material:**

The online version of this article (doi:10.1186/s12967-014-0256-4) contains supplementary material, which is available to authorized users.

## Background

Psoriasis is a common T-cell mediated autoimmune disease, affecting 2-3% of the world’s population. Genetic studies of psoriasis have identified over 40 susceptibility loci [1-3]. One of the interesting findings of these studies is the observation that many of these genetic variants involve genes that are known to play important roles in anti-viral defense mechanisms. Notable among these are IL28RA, IFIH1, DDX58 [3], and RNF114 [4]. IL28RA codes for the alpha subunit of IL-28 receptor and forms a complex with the IL-10 receptor, IL10RB. This receptor complex interacts with three closely related virus-induced cytokines, IL28A, IL28B and IL29 and plays role in antiviral defense [5]. IFIH1 has been known to play an important role in sensing viral nucleic acids and in activation of anti-viral immune responses. DDX58 encodes the RIG-I innate antiviral receptor, which recognizes cytosolic double stranded RNA. The exact function of RNF114 is not known but its paralog RNF125 is involved in ubiquitination of the innate anti-viral receptors, RIG-I and MDA5 [6]. Additionally, certain HLA alleles associated with psoriasis such as HLA-B*57 and HLA-B*27, have been associated with robust viral control of HIV-1 [7,8].

Physiological studies on psoriatic skin have also indicated a role of anti-viral restriction factors or anti-viral proteins (AVP) in psoriasis. A previous study has found that AVPs such as MX1, BST2, ISG15 and OAS2 are strongly elevated in the skin of psoriatic patients in comparison to healthy controls [9]. The authors further observed that IL29 might be responsible for the antiviral milieu in psoriasis as its expression correlated with AVP levels. Recently, we have performed whole transcriptome analysis of psoriasis skin and found that antiviral restriction factors are strongly upregulated in psoriatic skin and not in atopic dermatitis skin (manuscript in preparation). Altogether, these results suggest that psoriasis patients might have a strong cutaneous anti-viral immunity. However, the inciting factors and consequences of this anti-viral immunity are not known.

Possible triggers for psoriasis have been attributed to drugs [10] or pathogens such as bacteria and possibly virus [11]. Human endogenous retroviruses (HERVs) might play a role in triggering these anti-viral immune responses, since the role of HERVs in the pathogenesis of autoimmune diseases has generated considerable interest [12]. HERVs exist as proviruses in the human genome and consist of 5-10 kb of sequence encoding three genes, *Gag, Pol* and *Env*, flanked on both sides by long terminal repeats (LTR), which are 300-1200 nucleotides in length. They are estimated to have integrated into human genome starting 30-40 million years ago and as recently as 150,000 years ago [13]. Most HERVs have undergone significant mutational changes and are not thought to encode infectious virus, although there may be exceptions [14]. There are many families of HERVs but the most recent and widespread entrants into the human genome belong to the HERV-K family of endogenous retrovirus (HML-2, human MMTV like family), which are believed to have integrated into the human genome 200,000 to 5 million years ago [15,16]. Studies also report that it as one of the most transcriptionally active HERV in human genome [17,18].

Expression of HERV-K is known to be up-regulated in several diseases, including breast cancer, ovarian cancer, during HIV infection, and rheumatoid arthritis (RA), where an increase in viral mRNA and viral load of HERV-K was observed in active disease [19-23]. However, functional consequences of this expression are not known. It has been proposed that HERVs can trigger immune responses directly by acting as super-antigens, by encoding auto-antigens or by mimicking the self-proteins. Indeed, antibodies against HERVs are often observed in the sera of patients, resulting from an increase of transcriptional and translational activity [24,25]. Alternatively, HERVs could indirectly affect immune responses by influencing expression of genes, regulating immune responses, or facilitating tolerance.

The first evidence of role of HERVs in psoriasis came from the observation of retrovirus like particles from the skin of psoriasis patients [26]. Bessis *et al.* [27] observed that most of psoriasis patients showed positive immunofluorescence staining for HERV-E transmembrane envelope glycoprotein while only 15% of normal skin samples were positive. Furthermore, using a pan-retroviral detection system, it was observed that endogenous retroviral sequences for HERV-K, HERV-E and ERV-9 are expressed both in psoriatic and in normal skin [28]. These authors then used specific primers for ERV-9 and saw a significant increased expression in lesional skin compared to controls, but did not report quantitative measurements on HERV-K or HERV-E. However, the biological significance and the level of expression of HERV in psoriasis are not entirely known.

In the present study, we examined the humoral immune response against proteins coded by HERV-K Gag and Env gene in psoriasis patients and controls. We further used a sensitive approach, quantitative reverse transcription PCR (RT-qPCR), to measure the expression level of a comprehensive panel of HERV-K sequences (gag, env, pol and rec) in lesional and non-lesional skin from psoriatic patients compared to normal skin from healthy controls.

## Methods

### Patient enrollment and sample collection

Fourteen subjects with chronic, plaque psoriasis with affected body surface area > 10% and not on systemic medications were recruited from the UCSF Dermatology Department. All subjects provided written, informed consent for study participation under the approval of the local Institutional Review Boards. Five-millimeter punch biopsies were taken from the edge of a psoriatic plaque as well as from non-lesional skin located greater than 2 cm from any affected area. Twenty seven normal skin samples were obtained from healthy control surgical discard specimens. Skin samples were stored in RNALater (Ambion) at −80°C. Samples were mechanically homogenized using a Bio-Gen Pro 200 homogenizer and total RNA was extracted using the RNeasy mini kit (Qiagen) and a proteinase K digestion step was included in the manufacturer’s protocol. RNA was treated with DNAse at two steps, first during the extraction and secondly before converting RNA to cDNA. The quantity and quality of the RNA was assessed using a Nanodrop 8000 and in some cases by using an Agilent 2100 Bioanalyzer. For the ELISA, healthy donor sera were obtained from the Blood Center of the Pacific of San Francisco (n = 16). Psoriasis sera were obtained from subjects with chronic, plaque psoriasis recruited from the UCSF Dermatology Department.

### Recombinant proteins and peptides

HERV-K (HML-2) envelope transmemembrane protein (recTM), surface unit (recSU) and Gag capsid (recCA) recombinant proteins were obtained as previously described [24,29]. Furthermore, a set of 164 overlapping “15-mer” HERV-K (HML-2) Gag peptides (JPT Peptide Technologies, Berlin, Germany) were used to comprehensively map the antibody response. 5 positive peptides were identified as reacting with healthy donor sera: 16-KRIGKELKQ AGRKGN (Matrix), 58-GYPGMPPAPQGRAPY (p15), 81-GVKQYGPNSPYMRTL (Capsid), 117-SIADEKARKVIVELM (Capsid) and 137-KCYNCGQIGHLKKNC (Nucleocapsid, NC).

### ELISA

ELISA was adapted from Michaud *et al*. [24]. Briefly, 96 microtiter wells plate (Nunc-Immuno Plate MaxiSorp Surface) were coated for 1 hour at 37°C with peptides at 10 μg/ml in phosphate buffer saline (PBS) or overnight at 4°C with recombinant protein (GeneArt) at 5 μg/ml in PBS. Plates were then washed 3 times with 200 μL of PBS/0.05%-Tween 20 and blocked with 100 μL of blocking buffer [PBS/2.5%-Bovine Serum Albumin (BSA)] at room temperature (RT). The samples were diluted in blocking buffer and incubated 2 h at RT in duplicates. Plates were then washed 3 times with 200 μL of PBS/0.05%-Tween 20. An anti-human IgG or anti-human IgM horseradish peroxidase (HRP)-conjugated secondary antibody was diluted (1:1000 for IgG and 1:2000 for IgM) in blocking buffer and incubated at RT for 1 hour. Plates were then washed 6 times with 200 μL of PBS/0.05%-Tween 20 and incubated for 10 minutes with 100 μL of TMB (3,3′,5,5′-tetramethylbenzidine, Invitrogen). Addition of 50 μL H2SO_4_ 2 M stopped the reaction. The plates were read at 450 nm and 690 nm for the background on a plate reader. Background from 450 nm uncoated wells and PBS-BSA as negative controls was subtracted from the mean absorbance of the coated wells.

### Quantitative RT-PCR

Expression of HERV-K (Gag, Pol, Env and Rec) and ERV-9 were studied using previous published and validated primer sequences [30,31] (Additional file 1). DNAse treatment, cDNA conversion and PCR for all samples were done by Genomic Analysis Core Facility, Helen Diller Family Comprehensive Cancer Center, UCSF. Real time PCR was performed using the TaqMan chemistry on the ABI Prism 7900HT instrument (Life Technologies). PCR was conducted in triplicate with 20 μL reaction volumes of 1X Taqman buffer (1X Applied Biosystems PCR buffer, 20% glycerol, 2.5% gelatin, 60nM Rox as a passive reference), 5.5 mM MgCl2, 0.5 mM each primer, 0.2 μM each deoxynucleotide triphosphate (dNTP), 200 nM probe, and 0.025 unit/μL AmpliTaq Gold (Applied Biosystems) with 5 ng cDNA. A large master mix of the above-mentioned components (minus the primers, probe, and cDNA) was made for each experiment and aliquoted into individual tubes, one for each cDNA sample. cDNA was then added to the aliquoted master mix. The master mix with cDNA was aliquoted into a 384-well plate. The primers and probes were mixed together and added to the master mix and cDNA in the 384-well plate. PCR was conducted using the following cycle parameters: 1 cycle of 95° for 10 minutes and 40 cycles of 95° for 15 seconds, 60° for 1 minute. Analysis was carried out using the SDS software (version 2.3) supplied with the ABI 7900HT to determine the Cq values of each reaction. Cq values were determined for three test and three reference reactions in each sample, averaged, and subtracted to obtain the ΔCq [ΔCq = Cq (target) – Cq (reference)]. PCR efficiencies were measured for all custom assays and were greater than or equal to 90%.

RPLP0 (Taqman assay: Hs04189669_g1, Life Technologies) was used as the reference gene. The relative mRNA expression was measured as 2^(−ΔCq)*100 according to Schimttgen and Livak [32]. All the genes were compared independently.

### Statistical analyses

Humoral responses assayed by ELISA were compared between groups using the two-tailed Mann-Whitney tests. The relative mRNA was compared among the different groups using Anova Kruskal-Wallis and Dunn’s multiple comparison tests. All tests were conducted using GraphPad Prism, version 6.00 (GraphPad Software, San Diego, CA), with the statistical significance of the findings set at a p value of less than 0.05.

## Results

### Antibody responses to HERV-K proteins in psoriasis and control sera

We first investigated the humoral immune response against HERV-K in the sera of psoriasis patients and healthy controls. For these experiments, we examined the antibody responses against the two envelope sub-units encoded by the Env gene: the transmembrane protein (Env-recTM) and surface unit (Env-recSU), and the capsid encoded by Gag (Gag-recCA).

**Figure 1:**
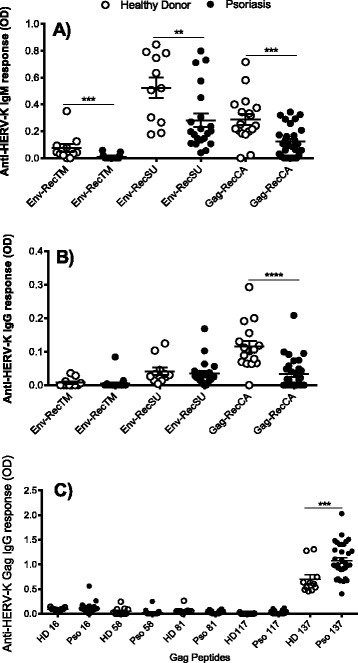
**Measurement of anti-HERV-K (HML-2) Env and Gag antibodies in psoriatic patients.** Graphs represent the detection of IgM against recombinant Env transmembrane (recTM), recombinant Env surface unit (recSU) and recombinant Gag capsid (recCA) proteins **(A)** or IgG against recTM, recSU and recCA **(B)** and individual HERV-K (HML-2) Gag peptides **(C)** in healthy donor (white dots, n = 16) and psoriatic (black dot, n = 28) sera. The figure shows the representative results of three independent experiments. In all graphs data represent mean and standard error of mean (SEM). A p value <0.05 was considered as significant. *p < 0.05, **p < 0.01, ***p < 0.001.

In healthy donors, we observed a basal level of IgM reacting with both Env-recSU and Gag-recCA while the response against Env-recTM was low. Interestingly, the IgM response was significantly decreased in psoriatic sera against all three recombinant proteins (Figure 1A). In healthy donor sera, the IgG response against HERV-K Env (SU and TM) was very low or absent, while a modest response against Gag-recCA was seen. This latter is significantly decreased in psoriasis patients in comparison to controls while the responses against Env-recSU and Env-recTM were not significantly modified in psoriasis patients (Figure 1B). Furthermore, we also examined IgG responses against five Gag peptides that we have previously identified as reacting with healthy donor sera among a set of 164 overlapping “15-mer” HERV-K (HML-2) Gag peptides (see Methods). We identified a significant increase of IgG in psoriatic sera against peptide 137, belonging to the HERV-K (HML-2) nucleocapsid, while the four other peptides did not display any significant difference in antibody levels (Figure 1C).

Together, these results demonstrated a significant reduction in IgM and IgG anti-HERV-K (HML-2) antibodies for most of the epitopes tested in psoriasis patients, suggesting the possibility that these proteins might be transcribed at a lower level in patients with psoriasis.

### Differential expression of HERV genes in psoriatic and normal skin

To investigate whether HERV antibody titers might be influenced by HERV expression levels, we performed RT-qPCR analysis on RNA obtained from lesional and non-lesional skin from a different set of psoriasis patients. We initially screened our samples for expression of 13 HERV genes representing several HERV families (HERV-K Gag, HERV-K Pol, HERV-K Env, ERV-9, HERV-K Rec, HERV-E4.1, HERV-R, HERV-H, HERV-L, HERV-W, HML-2, HML-4, HML-5) using published degenerate primer/probe sequences [30,31]. For our screen, we pooled cDNA from 11 lesional and 4 non-lesional samples and performed efficiency testing using five-fold serial dilutions of the cDNA pool. Of the 13 genes screened, only 5 (HERV-K Gag, HERV-K Pol, HERV-K Env, HERV-K Rec, ERV-9) were efficient. The other 8 genes were either not efficient or did not meet the minimal threshold for expression in our cDNA pool and thus were not further evaluated.

We evaluated expression of the remaining five genes (HERV-K Gag, HERV-K Pol, HERV-K Env, HERV-K Rec, ERV-9) by qPCR in skin biopsies from 14 psoriasis patients (non-lesional and lesional) and 27 healthy controls. We used RPLP0 as an endogenous control, as it has been validated as a reference gene in prior studies of psoriasis [33-36]. Furthermore, we confirmed that RPLP0 is not differentially expressed between psoriasis and controls by performing RNA sequencing on an independent set of psoriasis patients (18 lesional and 16 healthy skin controls), with a differential expression q-value of 0.123 (not significant).

**Figure 2:**
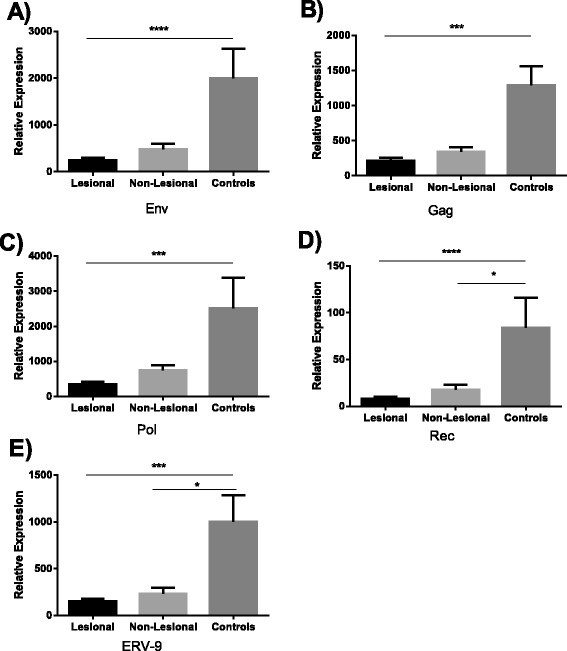
**Relative HERV mRNA expression of Env, Gag, Pol and Rec in psoriatic patients.** Graphs represent the expression of Env **(A)**, Gag **(B)**, Pol **(C)**, Rec **(D)** and ERV-9 **(E)** mRNA relative to that of reference gene RPLP0. All the experiments were done in triplicate. In all graphs data represent mean and SEM. Black bar represents lesional skin (n = 14), light grey bar indicates non-lesional skin (n = 14) and dark grey bar indicates healthy control skin (n = 27). A p value <0.05 was considered as significant. *p < 0.05, **p < 0.01, ***p < 0.001, ****p < 0.0001. In case of Rec, high Cq values were obtained.

We found that psoriasis patients and controls both showed detectable expression of HERV genes but that lesional skin from psoriasis patients showed a significant decrease in expression of each gene as compared to skin from healthy controls (Figure 2A to E). There was also a significant decrease in expression between psoriasis non-lesional skin and control for the HERV-K Rec and ERV-9 genes. The HERV-K Env, Gag and Pol genes also showed a trend towards decreased expression in non-lesional skin as compared to control skin, with p values of 0.08, 0.05, and 0.4, respectively.

## Discussion

Previous studies on HERVs indicate an association with several autoimmune diseases, cancers and even several infectious diseases. Some studies have also reported an association of HERVs and psoriasis [27,28]. However, these studies did not quantify the level of expression of HERV-K, which is known to have entered into human genome very recently and which is also believed to most functionally active of all endogenous retroviruses. To understand the association of HERV-K expression with psoriasis, we first examined the humoral immune response against HERV-K recombinant proteins in sera of psoriasis patients and controls. We observed that psoriasis patients showed a significant decrease in IgM responses against HERV-K Env-recTM, Env-recSU, and Gag-recCA. Coherent with this observation, the IgG response against those proteins were either not modified or decreased in psoriasis patients.

When we measured the responses against the more immunogenic linear peptide epitopes of HERV-K (HML-2) Gag (see Methods), we detected an increase in antibody titer against a single linear epitope belonging to the nucleocapsid (Gag 137). This epitope is also the most immunogenic in healthy donors. Although we detected a decreased antibody response against HERV-K recombinant proteins and an increased antibody response against Gag 137, discordant antibody responses against HERV-K (HML-2) have previously been reported. For instance, although being derived from a single transcript, SU and TM [37,38] show discordant transcriptional regulation in HIV-1 patients resulting in different specific humoral responses [24]. We can thus speculate that, as observed during HIV-1 infection, HERV-K (HML-2) transcriptional activity of different transcripts might be differentially modified during psoriasis. Alternatively, high specificity of B-cell receptor or MHC Class II binding to a particular epitope may lead to heightened antibody responses. Of note, we have previously shown that psoriasis patients have stronger antibody responses against HERV-K dUTPase recombinant protein compared to healthy controls [39]. It is noteworthy that only peptide 137 elicited responses in either controls or patients, and this 15mer has a 12/15 amino acid identity with the amino acid positions 74–88 of the predicted CaO19.10692 protein from *Candida albicans*, which is known to be more frequent in psoriasis patients [40] and could potentially cause cross reaction.

We further analyzed the expression of various HERV-families in a pooled set of cDNAs derived from 11 lesional and 4 non-lesional skin samples. However, we only observed expression of HERV-K genes (Env, Gag, Pol and Rec) and ERV-9. We then compared the expression level of HERV-K (Env, Gag, Pol and Rec) and ERV-9 genes in a larger set of patients, 14 pairs of lesional and non-lesional skin biopsies from psoriasis patients and 27 biopsies from healthy controls. In accordance with our ELISA findings, we observed a decreased expression of all HERV-K genes tested as well as ERV-9 in comparison to healthy controls.

Regarding the relationship of our ELISA results and our RT-qPCR results, there was a partial overlap between mRNA sequences examined by RT-qPCR and the proteins used for antibody testing. Our primer/probe set for the Env gene amplifies a part of SU gene, thus the decrease in Env expression seen by RT-qPCR directly correlates to the decrease in IgG and IgM responses against SU by ELISA. In contrast, our primer/probes for Gag amplify part of NC and thus do not directly correlate with the decreased antibody response seen here in case of CA. These results might be verified by either using primer/probes that amplify CA or using recombinant NC for analysis of antibody responses.

Taken together, our results demonstrate that decreased expression of HERV-K Gag and Env in psoriasis patients correlate with a decreased antibody response against these proteins. Although the decreased expression levels are not necessarily causal for the decreased antibody responses, the relationship is suggestive. Interestingly, a similar decrease in ERV expression was observed in a recent study on systemic lupus erythematosus (SLE) using RNA sequencing to characterize SLE transcriptome [41]. Thus, certain inflammatory diseases may be associated with suppression of HERV transcription.

There are several possible mechanisms to explain the observed decreased expression of HERV-K in psoriasis. It has been observed that global control of HERV expression can occur by heritable changes in gene expression without any change in underlying DNA sequences. These include alternation of DNA methylation or histone modification [42]. Past studies have reported that ERV transcription can be controlled by the methylation state of genomic DNA [43]. Furthermore, it has been shown previously that treatment of cells with agents promoting demethylation of genomic DNA could result in ERV induction [44]. All these findings have suggested that methylation of genomic DNA could be a way to control and regulate HERVs. Methylation studies done on psoriasis skin samples have observed differentially methylated regions (DMRs) covering a large part of the genome [45,46]. Furthermore, Zhang *et al.* [45] observed that the number of hypermethylated DMRs was considerably higher than that of hypomethylated DMRs in lesional samples form psoriasis patients. Whether these hypermethylated sites correspond to the genomic locations of HERVs would be interesting to determine.

Another mechanism that might affect HERV expression is RNA degradation of HERVs at the level of post-transcription. In fact, recent studies done in this regard indicate that control of HERVs can occur both at the level of transcript repression by methylation and RNA degradation at post-transcription and these two mechanisms can be interrelated. A third possibility is RNA interference. It has been speculated that dsRNA derived from retrotransposons and other retroelements may induce both transcript degradation and DNA methylation by using RNA interference (RNAi) pathways [43] and this can be driven by small interfering (siRNA) and Piwi-interacting RNA. Furthermore, these small RNAs can also help in targeting of repeats for DNA methylation and other chromatin modifications mechanisms.

Our present results contrast the results from Bessis *et al*., and Moles *et al*., [27,28] in that they found increased HERV expression and we have found decreased HERV expression. However, Bessis *et al*. only focused on HERV-E, and as mentioned earlier not all HERVs will be expressed in the same manner and this may account for a difference between HERV-E and HERV-K expression. Moles *et al*. did examine HERV-K sequences, but only measured ERV-9. Even so, we did find ERV-9 with a lower expression level in psoriasis patients, whereas they found a higher level of expression. More studies are needed to explain this discrepancy, but our ERV-9 data is consistent with our HERV-K results.

## Conclusions

In summary, we have shown that psoriasis patients show a decrease in antibody response to HERV-K proteins as compared to healthy controls. Furthermore, we also observed a decrease in expression of HERV-K genes Env, Gag, Pol and Rec and ERV-9 in psoriasis skin as compared to healthy skin. The reasons for the unexpected, low levels of HERV expression in psoriatic lesions are unclear and might be in part a consequence of anti-viral defense mechanisms. Whether these are triggered by HERV expression, expression of other antigens, and/or antigen independent mechanisms pose interesting questions for future study. Suppression of HERV-K expression across the genome may also indicate the possibility that the inflammatory state of psoriasis is associated with epigenetic changes leading to the observed decrease. However, further studies are warranted to explore this hypothesis.

### Study limitations

A limitation of this study is the moderate sample size examined. Some degree of variability was observed in mRNA expression across different genes. Independent replication of our findings in other cohorts is warranted.

## Additional file

Additional file 1:
**Primer/probe sequences of HERV genes tested in the study.**

## Abbreviations

HERV: Human Endogenous retrovirus
Gag: Group specific antigen
Pol: Polymerase
Env: Envelope
CA: Capsid
TM: Transmembrane
SU: Surface Unit
NC: Nucleocapsid
RT-qPCR: Reverse transcription-quantitative polymerase chain reaction
Cq: Quantification cycle
DMR: Differentially methylated regions
SLE: Systemic lupus erythematosus

## References

[1] Nair RP, Duffin KC, Helms C, Ding J, Stuart PE, Goldgar D, Gudjonsson JE, Li Y, Tejasvi T, Feng B-J, Ruether A, Schreiber S, Weichenthal M, Gladman D, Rahman P, Schrodi SJ, Prahalad S, Guthery SL, Fischer J, Liao W, Kwok PY, Menter A, Lathrop GM, Wise CA, Begovich AB, Voorhees JJ, Elder JT, Krueger GG, Bowcock AM, Abecasis GR (2009). Genome-wide scan reveals association of psoriasis with IL-23 and NF-κB pathways. Nat Genet.

[2] Strange A, Capon F, Spencer CC, Knight J, Weale ME, Allen MH, Barton A, Band G, Bellenguez C, Bergboer JG, Blackwell JM, Bramon E, Bumpstead SJ, Casas JP, Cork MJ, Corvin A, Deloukas P, Dilthey A, Duncanson A, Edkins S, Estivill X, Fitzgerald O, Freeman C, Giardina E, Gray E, Hofer A, Hüffmeier U, Hunt SE, Irvine AD, Genetic Analysis of Psoriasis C, the Wellcome Trust Case Control C (2010). A genome-wide association study identifies new psoriasis susceptibility loci and an interaction between HLA-C and ERAP1. Nat Genet.

[3] Tsoi LC, Spain SL, Knight J, Ellinghaus E, Stuart PE, Capon F, Ding J, Li Y, Tejasvi T, Gudjonsson JE, Kang HM, Allen MH, McManus R, Novelli G, Samuelsson L, Schalkwijk J, Ståhle M, Burden AD, Smith CH, Cork MJ, Estivill X, Bowcock AM, Krueger GG, Weger W, Worthington J, Tazi-Ahnini R, Nestle FO, Hayday A, Hoffmann P, Winkelmann J (2012). Identification of 15 new psoriasis susceptibility loci highlights the role of innate immunity. Nat Genet.

[4] Bijlmakers MJ, Kanneganti SK, Barker JN, Trembath RC, Capon F (2011). Functional analysis of the RNF114 psoriasis susceptibility gene implicates innate immune responses to double-stranded RNA in disease pathogenesis. Hum Mol Genet.

[5] Sheppard P, Kindsvogel W, Xu W, Henderson K, Schlutsmeyer S, Whitmore TE, Kuestner R, Garrigues U, Birks C, Roraback J, Ostrander C, Dong D, Shin J, Presnell S, Fox B, Haldeman B, Cooper E, Taft D, Gilbert T, Grant FJ, Tackett M, Krivan W, McKnight G, Clegg C, Foster D, Klucher KM (2003). IL-28, IL-29 and their class II cytokine receptor IL-28R. Nat Immunol.

[6] Arimoto K, Takahashi H, Hishiki T, Konishi H, Fujita T, Shimotohno K (2007). Negative regulation of the RIG-I signaling by the ubiquitin ligase RNF125. Proc Natl Acad Sci U S A.

[7] Chen H, Hayashi G, Lai OY, Dilthey A, Kuebler PJ, Wong TV, Martin MP, Fernandez Vina MA, McVean G, Wabl M, Leslie KS, Maurer T, Martin JN, Deeks SG, Carrington M, Bowcock AM, Nixon DF, Liao W (2012). Psoriasis patients are enriched for genetic variants that protect against HIV-1 disease. PLoS Genet.

[8] International HIVCS, Pereyra F, Jia X, McLaren PJ, Telenti A, de Bakker PI, Walker BD, Ripke S, Brumme CJ, Pulit SL, Carrington M, Kadie CM, Carlson JM, Heckerman D, Graham RR, Plenge RM, Deeks SG, Gianniny L, Crawford G, Sullivan J, Gonzalez E, Davies L, Camargo A, Moore JM, Beattie N, Gupta S, Crenshaw A, Burtt NP, Guiducci C, Gupta N (2010). The major genetic determinants of HIV-1 control affect HLA class I peptide presentation. Science.

[9] Wolk K, Witte K, Witte E, Raftery M, Kokolakis G, Philipp S, Schonrich G, Warszawska K, Kirsch S, Prosch S, Sterry W, Volk HD, Sabat R (2013). IL-29 is produced by T(H)17 cells and mediates the cutaneous antiviral competence in psoriasis. Sci Transl Med.

[10] Rongioletti F, Fiorucci C, Parodi A (2009). Psoriasis induced or aggravated by drugs. J Rheumatol Suppl.

[11] Fry L, Baker BS (2007). Triggering psoriasis: the role of infections and medications. Clin Dermatol.

[12] Tugnet N, Rylance P, Roden D, Trela M, Nelson P (2013). Human Endogenous Retroviruses (HERVs) and Autoimmune Rheumatic Disease: Is There a Link?. Open Rheumatol J.

[13] Jha AR, Nixon DF, Rosenberg MG, Martin JN, Deeks SG, Hudson RR, Garrison KE, Pillai SK (2011). Human endogenous retrovirus K106 (HERV-K106) was infectious after the emergence of anatomically modern humans. PLoS One.

[14] Dewannieux M, Harper F, Richaud A, Letzelter C, Ribet D, Pierron G, Heidmann T (2006). Identification of an infectious progenitor for the multiple-copy HERV-K human endogenous retroelements. Genome Res.

[15] Barbulescu M, Turner G, Seaman MI, Deinard AS, Kidd KK, Lenz J (1999). Many human endogenous retrovirus K (HERV-K) proviruses are unique to humans. Curr Biol.

[16] Turner G, Barbulescu M, Su M, Jensen-Seaman MI, Kidd KK, Lenz J (2001). Insertional polymorphisms of full-length endogenous retroviruses in humans. Curr Biol.

[17] Sugimoto J, Matsuura N, Kinjo Y, Takasu N, Oda T, Jinno Y (2001). Transcriptionally active HERV-K genes: identification, isolation, and chromosomal mapping. Genomics.

[18] Tonjes RR, Lower R, Boller K, Denner J, Hasenmaier B, Kirsch H, Konig H, Korbmacher C, Limbach C, Lugert R, Phelps RC, Scherer J, Thelen K, Löwer J, Kurth R (1996). HERV-K: the biologically most active human endogenous retrovirus family. J Acquir Immune Defic Syndr Hum Retrovirol.

[19] Contreras-Galindo R, Kaplan MH, Leissner P, Verjat T, Ferlenghi I, Bagnoli F, Giusti F, Dosik MH, Hayes DF, Gitlin SD, Markovitz DM (2008). Human endogenous retrovirus K (HML-2) elements in the plasma of people with lymphoma and breast cancer. J Virol.

[20] Contreras-Galindo R, Kaplan MH, Contreras-Galindo AC, Gonzalez-Hernandez MJ, Ferlenghi I, Giusti F, Lorenzo E, Gitlin SD, Dosik MH, Yamamura Y, Markovitz DM (2012). Characterization of human endogenous retroviral elements in the blood of HIV-1-infected individuals. J Virol.

[21] Depil S, Roche C, Dussart P, Prin L (2002). Expression of a human endogenous retrovirus, HERV-K, in the blood cells of leukemia patients. Leukemia.

[22] Freimanis G, Hooley P, Ejtehadi HD, Ali HA, Veitch A, Rylance PB, Alawi A, Axford J, Nevill A, Murray PG, Nelson PN (2010). A role for human endogenous retrovirus-K (HML-2) in rheumatoid arthritis: investigating mechanisms of pathogenesis. Clin Exp Immunol.

[23] Reynier F, Verjat T, Turrel F, Imbert PE, Marotte H, Mougin B, Miossec P (2009). Increase in human endogenous retrovirus HERV-K (HML-2) viral load in active rheumatoid arthritis. Scand J Immunol.

[24] Michaud HA, de Mulder M, SenGupta D, Deeks SG, Martin JN, Pilcher CD, Hecht FM, Sacha JB, Nixon DF (2014). Trans-activation, post-transcriptional maturation, and induction of antibodies to HERV-K (HML-2) envelope transmembrane protein in HIV-1 infection. Retrovirology.

[25] Wang-Johanning F, Radvanyi L, Rycaj K, Plummer JB, Yan P, Sastry KJ, Piyathilake CJ, Hunt KK, Johanning GL (2008). Human endogenous retrovirus K triggers an antigen-specific immune response in breast cancer patients. Cancer Res.

[26] Dalen AB, Hellgren L, Iversen OJ, Vincent J (1983). A virus-like particle associated with psoriasis. Acta Pathol Microbiol Immunol Scand B.

[27] Bessis D, Moles JP, Basset-Seguin N, Tesniere A, Arpin C, Guilhou JJ (2004). Differential expression of a human endogenous retrovirus E transmembrane envelope glycoprotein in normal, psoriatic and atopic dermatitis human skin. Br J Dermatol.

[28] Moles JP, Tesniere A, Guilhou JJ (2005). A new endogenous retroviral sequence is expressed in skin of patients with psoriasis. Br J Dermatol.

[29] George M, Schwecke T, Beimforde N, Hohn O, Chudak C, Zimmermann A, Kurth R, Naumann D, Bannert N (2011). Identification of the protease cleavage sites in a reconstituted Gag polyprotein of an HERV-K(HML-2) element. Retrovirology.

[30] Jones RB, Garrison KE, Mujib S, Mihajlovic V, Aidarus N, Hunter DV, Martin E, John VM, Zhan W, Faruk NF, Gyenes G, Sheppard NC, Priumboom-Brees IM, Goodwin DA, Chen L, Rieger M, Muscat-King S, Loudon PT, Stanley C, Holditch SJ, Wong JC, Clayton K, Duan E, Song H, Xu Y, SenGupta D, Tandon R, Sacha JB, Brockman MA, Benko E (2012). HERV-K-specific T cells eliminate diverse HIV-1/2 and SIV primary isolates. J Clin Invest.

[31] Pichon JP, Bonnaud B, Mallet F (2006). Quantitative multiplex degenerate PCR for human endogenous retrovirus expression profiling. Nat Protoc.

[32] Schmittgen TD, Livak KJ (2008). Analyzing real-time PCR data by the comparative C(T) method. Nat Protoc.

[33] Wingens M, van Bergen BH, Hiemstra PS, Meis JF, van Vlijmen-Willems IM, Zeeuwen PL, Mulder J, Kramps HA, van Ruissen F, Schalkwijk J (1998). Induction of SLPI (ALP/HUSI-I) in epidermal keratinocytes. J Invest Dermatol.

[34] Mitsui H, Suarez-Farinas M, Belkin DA, Levenkova N, Fuentes-Duculan J, Coats I, Fujita H, Krueger JG (2012). Combined use of laser capture microdissection and cDNA microarray analysis identifies locally expressed disease-related genes in focal regions of psoriasis vulgaris skin lesions. J Invest Dermatol.

[35] Suarez-Farinas M, Lowes MA, Zaba LC, Krueger JG (2010). Evaluation of the psoriasis transcriptome across different studies by gene set enrichment analysis (GSEA). PLoS One.

[36] Zaba LC, Cardinale I, Gilleaudeau P, Sullivan-Whalen M, Suarez-Farinas M, Fuentes-Duculan J, Novitskaya I, Khatcherian A, Bluth MJ, Lowes MA, Krueger JG (2007). Amelioration of epidermal hyperplasia by TNF inhibition is associated with reduced Th17 responses. J Exp Med.

[37] Dewannieux M, Blaise S, Heidmann T (2005). Identification of a functional envelope protein from the HERV-K family of human endogenous retroviruses. J Virol.

[38] Hanke K, Kramer P, Seeher S, Beimforde N, Kurth R, Bannert N (2009). Reconstitution of the ancestral glycoprotein of human endogenous retrovirus k and modulation of its functional activity by truncation of the cytoplasmic domain. J Virol.

[39] Lai OY, Chen H, Michaud HA, Hayashi G, Kuebler PJ, Hultman GK, Ariza ME, Williams MV, Batista MD, Nixon DF, Foerster J, Bowcock AM, Liao W (2012). Protective effect of human endogenous retrovirus K dUTPase variants on psoriasis susceptibility. J Invest Dermatol.

[40] Waldman A, Gilhar A, Duek L, Berdicevsky I (2001). Incidence of Candida in psoriasis–a study on the fungal flora of psoriatic patients. Mycoses.

[41] Shi L, Zhang Z, Yu AM, Wang W, Wei Z, Akhter E, Maurer K, Reis PC, Song L, Petri M, Sullivan KE (2014). The SLE Transcriptome Exhibits Evidence of Chronic Endotoxin Exposure and Has Widespread Dysregulation of Non-Coding and Coding RNAs. PLoS One.

[42] Stoye JP (2012). Studies of endogenous retroviruses reveal a continuing evolutionary saga. Nat Rev Microbiol.

[43] Maksakova IA, Mager DL, Reiss D (2008). Keeping active endogenous retroviral-like elements in check: the epigenetic perspective. Cell Mol Life Sci.

[44] Groudine M, Eisenman R, Weintraub H (1981). Chromatin structure of endogenous retroviral genes and activation by an inhibitor of DNA methylation. Nature.

[45] Zhang P, Zhao M, Liang G, Yin G, Huang D, Su F, Zhai H, Wang L, Su Y, Lu Q (2013). Whole-genome DNA methylation in skin lesions from patients with psoriasis vulgaris. J Autoimmun.

[46] Roberson ED, Liu Y, Ryan C, Joyce CE, Duan S, Cao L, Martin A, Liao W, Menter A, Bowcock AM (2012). A subset of methylated CpG sites differentiate psoriatic from normal skin. J Invest Dermatol.

